# Novel use of synthetic peptide hemostatic gel in conjunction with EUS-guided coil injection for the treatment of gastric varices: A single-center experience

**DOI:** 10.1097/eus.0000000000000179

**Published:** 2026-08-21

**Authors:** Christina S. Gainey, Andrew Canakis, Todd H. Baron

**Affiliations:** Division of Gastroenterology and Hepatology, University of North Carolina at Chapel Hill, Chapel Hill, NC, USA.

**Keywords:** Endoscopic ultrasound, Gastric varices, Variceal bleed, Therapeutic endoscopic ultrasound, Hemostatic agents

## Abstract

**Background and Objectives:**

Bleeding from gastric varices (GV) is severe and associated with high mortality. The standard endoscopic therapy, EUS-guided coil injection with cyanoacrylate glue (EUS-COIL + glue), is highly effective but carries a risk of systemic embolization. This study investigates the safety and efficacy of PuraStat, a synthetic peptide hemostatic gel that promotes clot formation without embolization risk, as an alternative injectate in conjunction with EUS-COIL.

**Methods:**

This was a single-center, retrospective analysis of 9 patients who underwent EUS-COIL + PuraStat for GV treatment (active bleeding, recent bleeding, or primary prophylaxis). Technical success was defined as successful coil deployment and reduction of Doppler flow; clinical success as bleeding cessation and/or absence of bleeding at 30 days. Secondary outcomes included adverse events (AEs) and re-intervention rates during follow-up.

**Results:**

Technical and clinical success was achieved in 100% of the procedures. There were no intraprocedural AEs, and only 1 minor (Grade II) postprocedural AE was recorded (10%). No patients experienced systemic embolization. One patient (11%) experienced recurrent bleeding at 54 days. Over a mean follow-up of 289 days, 56% of patients required repeat EUS-COIL procedures.

**Conclusion:**

EUS-COIL with PuraStat is a safe and highly effective treatment for GV in this small cohort, demonstrating excellent immediate success without the risk of systemic embolization associated with cyanoacrylate glue. While its use may necessitate earlier and more frequent surveillance and re-intervention, its superior safety profile makes it an attractive option. Larger comparative studies are warranted.

## INTRODUCTIONS

Bleeding from gastric varices (GV) is less common than esophageal varices, but tends to be more severe, with higher rates of treatment failure, rebleeding, and mortality.^[1]^ GVs are most commonly caused by portal hypertension due to cirrhosis, but are also more common in those with prehepatic portal hypertension, such as from isolated splenic vein thrombosis (e.g., from pancreatic disease) or portal vein thrombosis. The 3-year estimated incidence of bleeding from cardiofundal GV is 16%–45%.^[1]^

In patients with acute hemorrhage from gastrofundal varices, endoscopic cyanoacrylate injection, transjugular intrahepatic portosystemic shunt (TIPS) procedure, or retrograde transvenous variceal embolization/obliteration can be considered first-line options to control bleeding and prevent rebleeding. The determination depends on anatomy, presence of portosystemic shunts, and other portal hypertensive complications.

EUS-guided coil injection therapy (EUS-COIL) with cyanoacrylate (glue) injection is a highly effective treatment for GV. While cyanoacrylate glue rapidly seals the vessel, the coils act as a scaffold for clot formation and to hold the glue in place, maximizing hemostasis and potentially reducing the rate of embolization.^[2]^ This combined approach has a high success rate (96%–98%) and a low adverse event (AE) rate (10%)^[3–5]^ and is considered the preferred strategy by experts.

While combining coils with cyanoacrylate can reduce the risk of systemic embolization, it does not eliminate it completely. While most cases are asymptomatic, embolization can result in catastrophic pulmonary embolism and stroke.

A potential alternative to glue is the synthetic peptide hemostatic gel, PuraStat (3D Matrix Europe SAS, France), which has a key advantage: it does not appear to carry a risk of embolization. When it comes into contact with blood or tissue fluids, it rapidly forms a three-dimensional hydrogel that acts as a physical barrier to promote clot formation. A 2023 meta-analysis found it to be highly effective for treating gastrointestinal bleeding (93.1% success) with low rebleeding rates (8.9%).^[6]^ This is the first retrospective analysis to describe the use of synthetic peptide hemostatic gel, PuraStat, in conjunction with EUS-COIL for the treatment of GV.

## METHODS

This is a single-center retrospective, database study evaluating patients who had an EUS-COIL + PuraStat from January 1, 2014, to September 4, 2025. Our center began using PuraStat instead of glue starting in July 2023.

All procedures were conducted by a single experienced endoscopist. Baseline patient information, indications for procedure, and procedure details were recorded. This study was approved by the institutional review board.

Primary outcomes included technical and clinical success. Technical success was defined by the successful deployment of the coil into the varix under EUS guidance with diminution of Doppler flow. Clinical success was defined as cessation of bleeding, if present, and/or absence of bleeding at 30 days postintervention. Secondary outcomes included total AEs, rates of recurrent bleeding, re-intervention rates, mean time to EUS surveillance, GV obliteration or reduction in Doppler flow at surveillance, and death during follow-up.

Procedure-related AEs were defined as early (within 30 days) or late (after 30 days). AEs were recorded for the index procedure and repeat procedures. AE severity was graded according to the American Society for Gastrointestinal Endoscopy AGREE classification.^[7]^

### Statistical analysis

Categorical variables were reported as percentages, and quantitative variables were reported as mean ± standard deviation (SD). Statistical analysis was performed using Microsoft Excel 2024 (Microsoft Corporation, Redmond, Wash).

### Procedural technique

Initial endosonographic evaluation of GV with Doppler was performed using a standard oblique-viewing linear echoendoscope (Olympus, GF-UCT 180). A standard 19-gauge FNA needle (Expect, BSCI, Marlborough, MA) primed with normal saline was inserted into the varix under sonographic guidance. Embolization coils (Nester, Cook Medical) were inserted through the needle to fill the varix, followed by injection of 3–6 mL of synthetic peptide hemostatic gel, PuraStat (3D Matrix Europe SAS, Caluire-et-Cuire, France). The GV was again viewed with EUS to ensure the lack of Doppler flow.

## RESULTS

Nine patients were included in the study (**Table 1**). The mean age was 58 ± 17 years, 44% were female, and the mean American Society of Anesthesiologists classification was 3 ± 0.5. Four patients had underlying cirrhosis as the etiology of their GV, 4 patients had noncirrhotic portal hypertension, and 1 patient had chronic pancreatitis with a splenic vein thrombus. Two patients had had prior interventional radiology-guided interventions, including a balloon-occluded retrograde transvenous obliteration procedure and TIPS with surgical mesocaval shunt.

**TABLE 1:** Patient demographics

	Total (*N* = 9)
Age at procedure, yr (mean ± SD)	58 ± 17
Female, *n* (%)	4 (44)
ASA Classification (mean ± SD)	3 ± 0.5
Anticoagulation use, *n* (%)	3 (33)
Etiology of gastric varices, *n* (%)	
Cirrhosis	4 (44)
Alcohol	3
MASLD	1
Noncirrhotic portal HTN	4 (44)
Chronic portal vein thombus	3
Polycythemia vera	1
Chronic pancreatitis with splenic vein thrombus	1 (11)
Prior IR procedure, *n* (%)	2 (22)
BRTO	1
TIPS, surgical mesocaval shunt	1

ASA, American Society of Anesthesiologists; BRTO, balloon-occluded retrograde transvenous obliteration; HTN, hypertension; IR, interventional radiology; MASLD, metabolic dysfunction-associated steatotic liver disease; TIPS, transjugular intrahepatic portosystemic shunt.

Two procedures (22%) were performed for primary prophylaxis of bleeding (Table 2); 1 patient was not a candidate for balloon-occluded retrograde transvenous obliteration or TIPS procedure, and the other patient was intolerant to beta blockers due to hypotension. Six procedures (67%) were performed to treat active bleeding, and 1 was performed to treat GV that had recently bled but without ongoing bleeding. The majority of cases were performed inpatient (7/9, 78%). The mean size of varices was 7.7 ± 2.4 mm, and the mean number of coils used was 2.6 ± 1.5.

**TABLE 2:** Procedure details

	Total (*N* = 9)
Indication for EUS procedure, *n* (%)	
Primary prophylaxis	2 (22)
Treatment of active bleeding	6 (67)
Treatment of recent bleeding	1 (11)
Outpatient	2 (22)
Size of varices, mm (mean ± SD)	7.7 ± 2.4
Number of coils used (mean ± SD)	2.6 ± 1.5

Technical and clinical success were achieved in 100% of cases (Table 3). There were no intraprocedural adverse events. The only postprocedural AE (grade II) occurred 4 days after EUS-COIL when the patient presented with abdominal pain and fevers. The patient was admitted for 2 days, but quickly improved with no etiology found for the presentation.

**TABLE 3:** Outcomes

	Total (*N* = 9)
Technical success, *n* (%)	9 (100)
Clinical success, *n* (%)	9 (100)
Total adverse events, *n* (%)	1 (11)
Abdominal pain, fevers	1
Recurrent bleeding rate (%)	1 (11)
Death during follow-up (%)	2
Follow-up, days (mean ± SD)	289 ± 296
Surveillance EUS, *n* (%)	5 (56)
Time to surveillance EUS, days (mean ± SD)	182 ± 163
GV obliteration or reduction in Doppler flow at first surveillance	2/5 (40)
Number of repeat EUS procedures (mean ± SD)	1.6 ± 1.9
Number of repeat EUS procedures with intervention (mean ± SD)	0.9 ± 1.1
Re-intervention with EUS-COIL	5 (56)
Days to first repeat EUS-COIL (mean ± SD)	233 ± 238

EUS-COIL, EUS-guided coil injection therapy; GV, gastric varices.

Recurrent bleeding occurred in 1 patient at 54 days postintervention and was treated with EUS-COIL with injection of histoacryl and lipiodol mixture.

Two patients died during follow-up after transitioning to hospice; 1 for progressive hepatocellular carcinoma and the other for renal failure. Neither were related to complications from GV or interventions.

Mean follow-up time was 289 ± 296 days. Surveillance EUS was performed in 5 patients (56%) at a mean of 182 ± 163 days. Two other patients had follow-up EGDs with diminutive GV and no bleeding. At first, EUS surveillance, 2/5 patients (40%) had complete GV obliteration or reduction in Doppler flow. Patient underwent a mean of 1.6 ± 1.9 subsequent EUS procedures. The mean number of repeat EUS procedures with intervention was 0.9 ± 1.1. Around 5/9 patients (56%) underwent repeat EUS-COIL; the mean time to first repeat EUS-COIL was 233 ± 238 days.

## DISCUSSION

This study demonstrates that EUS-COIL with PuraStat injection is an effective and safe method for managing GV, achieving 100% technical and clinical success in our cohort of 9 patients. The procedure was successful both for primary prophylaxis in high-risk patients unsuitable for other therapies and, more critically, for the management of active or recent variceal bleeding.

A key strength of this approach is its excellent safety profile. We observed no intraprocedural AEs and only 1 minor, self-resolving postprocedural adverse event. Notably, unlike cyanoacrylate glue injections, which carry a known risk of systemic embolization. None of our patients has experienced this with PuraStat.

Our patient cohort was heterogeneous, with half of the patients having cirrhosis and the other half having noncirrhotic portal hypertension. A third of the patients were on anticoagulation for portal vein thrombosis, which may have contributed to a higher underlying risk of rebleeding.

Despite the high initial success rate, complete obliteration required further intervention in many cases. Over half of the patients (56%) underwent a repeat EUS procedure for re-treatment during a mean follow-up of nearly 10 months. While it is difficult to make comparisons given our small sample size, our re-intervention rate appears higher than the 12%–21% reported for EUS-COIL combined with cyanoacrylate glue,^[4,5,8,9]^ but it is comparable to the 50%–54% rate reported for EUS-COIL used alone.^[8,9]^ This finding suggests that its mechanism—creating a scaffold for natural clot formation rather than providing the immediate mechanical occlusion of glue—may result in less durable single-session obliteration. While it may require more frequent re-intervention compared with glue-based therapies, its superior safety profile makes it an attractive option.

Our study demonstrated the potential need for earlier surveillance EUS after initial treatment, particularly in high-risk patients. One patient experienced rebleeding 2 months after the initial procedure while on anticoagulation. Surveillance EUS revealed that complete obliteration was not achieved in the majority of patients after a single session, with only 40% showing complete obliteration or significant flow reduction at the first follow-up around the 6-month mark. This underscores the potential need for earlier surveillance EUS after the initial treatment, particularly in high-risk patients, to assess the adequacy of obliteration and intervene before rebleeding occurs.

This study has several important limitations that must be acknowledged. As a single-center retrospective analysis, the level of evidence remains exploratory. The lack of a control group prevents us from making definitive claims regarding superior efficacy or comparative cost-effectiveness. Furthermore, the small cohort (N = 9) restricts the generalizability of our findings.

In conclusion, EUS-COIL with PuraStat is a safe and highly effective treatment for GV, demonstrating excellent immediate outcomes for both bleeding cessation and prophylaxis in a small cohort of patients. However, in the absence of controlled studies, these findings should be viewed as preliminary. Larger future studies may further clarify its use.

## Ethical Statements

This study was approved by the Institutional Review Board at the University of North Carolina at Chapel Hill (IRB #24-1703). A waiver of informed consent was granted given the retrospective nature of the study.

## Conflicts of Interest

Dr. Baron is a consultant and speaker for Boston Scientific, W.L. Gore, Conmed, and GIE Medical. The remaining authors declare that they have no financial conflict of interest with regard to the content of this report.

## Author contribution

T. H. Baron conceived the study and contributed to manuscript editing and critical revision. C. S. Gainey drafted the manuscript, collected and organized the data, and edited the manuscript. A. Canakis contributed to critical revision. All authors approved the final version of the manuscript.

## Use of Large Language Models, AI and Machine Learning Tools

None declared.

## Data Availability Statement

None declared.
